# Domain-aware domain–class adaptation network for motor execution to motor imagery EEG classification

**DOI:** 10.3389/fnins.2026.1851006

**Published:** 2026-06-01

**Authors:** Jiahuan Wang, Guanghua Xu, Chenghang Du, Zejin Li, Hui Li, Shengchao Chen, Chengcheng Han, Sicong Zhang

**Affiliations:** 1 School of Mechanical Engineering, Xi’an Jiaotong University, Xi’an, China; 2 State Key Laboratory for Manufacturing Systems Engineering, Xi’an Jiaotong University, Xi’an, China; 3 The First Affiliated Hospital of Xi’an Jiaotong University, Xi’an, China; 4 State Industry-Education Integration Center for Medical Innovations, Xi’an Jiaotong University, Xi’an, China

**Keywords:** brain-computer interface, domain adaptation, electroencephalogram, motor execution, motor imagery, transfer learning

## Abstract

**Introduction:**

Motor imagery (MI) is one of the most widely used paradigms in electroencephalogram (EEG)-based brain–computer interfaces (BCIs). In recent years, deep learning and transfer learning techniques have been increasingly adopted to further improve MI-EEG decoding performance, thereby facilitating the practical deployment of BCIs. In transfer learning, the similarity between the source and target domains is a critical factor influencing its effectiveness. Given the analogous cortical activation patterns observed in MI and motor execution (ME) tasks, cross-task transfer learning from ME to MI presents a promising yet underexplored direction.

**Methods:**

To tackle the underexplored problem of cross-task transfer learning from ME to MI, we propose a domain-aware domain–class adaptation network (DDCA Net), which consists of a domain-shared feature extractor, two classifiers, and two domain-specific feature re-weighting blocks. Domain-level alignment is achieved by minimizing the maximum mean discrepancy between source and target feature distributions, while domain-specific feature re-weighting preserves discriminative characteristics unique to each task. In addition, a bi-classifier adversarial learning framework is employed to encourage consistency of decision boundaries across domains, thereby enabling implicit class-level alignment.

**Results:**

Extensive experiments were conducted on a public dataset with over 100 subjects under varying proportions of target-domain training samples. When 80% of target-domain samples are used for training, the proposed DDCA Net significantly outperforms the within-task baseline, achieving a 7.71% improvement in classification accuracy and converting approximately 80% of previously BCI-illiterate subjects into BCI-literate users.

**Discussion:**

To the best of our knowledge, this is the first work to verify the feasibility of applying domain adaptation for cross-task transfer learning in MI-EEG classification. The findings of this study provide new insights for integrating ME and MI in advanced BCIs.

## 1 Introduction

Electroencephalogram (EEG)-based Brain-computer interfaces (BCIs) establish a direct access between users and external devices without the participation of peripheral nerves and muscles (Saibene et al., 2024). EEG-based BCIs have been utilized not only in rehabilitation of patients with motor obstacle, but also in daily life assistance for healthy users. Motor imagery (MI) is imagining certain movement of the body part but without any actual motion (Li et al., 2019). As a representative of the endogenous and spontaneous BCI paradigm, MI has long been a focus of extensive research (Kim et al., 2022; Phadikar et al., 2023). The classification results of EEG during MI tasks will be sent to external devices as control signals in MI-BCI systems. Accurate classification of different movement types is essential to ensure external devices receive the correct instructions.

Early MI classification relied on conventional machine learning methods for feature extraction, which often utilize information insufficiently. In comparison, deep learning (DL) approaches enable automatic feature extraction and classification, demonstrating superior robustness and generalization ability. Several representative DL models have achieved strong performance in EEG decoding (Wang et al., 2024). For instance, both deep and shallow convolutional neural networks have achieved classification performance comparable to conventional machine learning methods (Schirrmeister et al., 2017). EEGNet has further demonstrated strong capability in decoding various BCI paradigms (Lawhern et al., 2018). In addition, numerous DL-based approaches have been proposed to further enhance EEG decoding performance (Zhang et al., 2020; Riyad et al., 2021; Hou et al., 2022).

In practice, it is challenging to develop a universal DL-based model that generalizes well across different subjects or recording sessions. Transfer learning (TL) has emerged as an effective solution, enabling the reuse of transferable knowledge from a source domain to improve prediction in a target domain based on their underlying similarity (Wu et al., 2022). In MI-EEG decoding, TL is commonly applied in cross-subject, cross-session, and cross-dataset scenarios.

In the cross-subject setting, EEG data from a specific subject performing a given MI task are treated as the target domain, while data from other subjects performing the same task serve as the source domain. The goal is to mitigate inter-subject variability and thereby improve the prediction accuracy for the target subject (Zhang and Wu, 2023). Zhang et al. (2021) and Roy (2022) investigated the influence of different pre-training–fine-tuning configurations on cross-subject TL in binary and quaternary MI-EEG classification. Besides fine-tuning, some domain adaptation (DA) methods were successfully applied in cross-subject TL. For example, DRDA (Zhao et al., 2021) and CDAN (Tang and Zhang, 2020) introduced domain discriminator to align source domain and target domain in an implicit and dynamic mode. Selective-MDA (Lee et al., 2024) and CLUDA (Xu et al., 2025) further optimize the DA process considering the discrepancies among subjects in source domain. In addition to cross-subject TL, cross-session TL (Hong et al., 2021; Zheng and Yang, 2021; Zhang et al., 2024) and cross-dataset TL (Wang et al., 2025; Chen et al., 2025) have been studied to address domain shifts caused by variations in the mental states of a specific subject and discrepancies across different EEG acquisition devices.

As discussed above, cross-subject, cross-session, and cross-dataset TL have been proposed to address practical challenges in real-world applications. From a TL perspective, the degree of similarity between the source and target tasks plays a crucial role in the effectiveness of knowledge transfer and, consequently, the performance on the target task. Given the inherent similarity between MI and motor execution (ME) tasks, cross-task TL from ME to MI has attracted increasing attention. Specifically, MI can be regarded as the covert style of ME. The neural activation of the motor cortex and EEG rhythm generated during MI and ME tasks are similar. It has been known that the amplitude characteristic and decoding performance of ME are stronger than those of MI. Comparative studies between MI and ME have reported that ME typically achieves higher classification accuracy than MI (Tang et al., 2016; Lashgari et al., 2021). Three possible explanations have been suggested for this phenomenon. First, ME signals tend to have stronger amplitude features, which mitigate the difficulty of signal decoding. Second, due to the presence of explicit movement, the consistency between the acquired signals and the intended commands during ME tasks is generally more convincible (Gwon and Ahn, 2024; Pérez-Velasco et al., 2025). Third, the duration of the task state cannot be measured accurately during MI tasks. As a result, the labels associated with MI are to some extent affected by noise. Furthermore, to advance the application of BCIs, it is essential to ensure that training data collection task is intuitive and user-friendly to minimize the users’ fatigue and stress. Compared to MI tasks, ME tasks have shown to be less challenging and demanding. Therefore, integrating MI with ME tasks during data acquisition and calibration may facilitate the development of more user-friendly BCI systems.

While TL techniques have been extensively investigated in cross-subject, cross-session, and cross-dataset scenarios, these studies are largely confined to intra-task settings. To date, cross-task TL for MI classification has received very limited attention. Lee et al. (2020) conducted a cross-task TL experiment and demonstrated the feasibility of proposed method; however, the study was limited to only nine participants. Miao et al. (2023) and Pérez-Velasco et al. (2025) evaluated cross-task transferability using public datasets. Since both cross-subject and cross-task settings were involved, the feasibility of cross-task TL on a specific subject remains uncertain. Gwon and Ahn (2024) comprehensively investigated the TL performance among ME, MI, and motor observation tasks. The possibility of cross-task was demonstrated when partial samples were available in target tasks, yet the TL method they employed lacked rigorous design.

**FIGURE 1:**
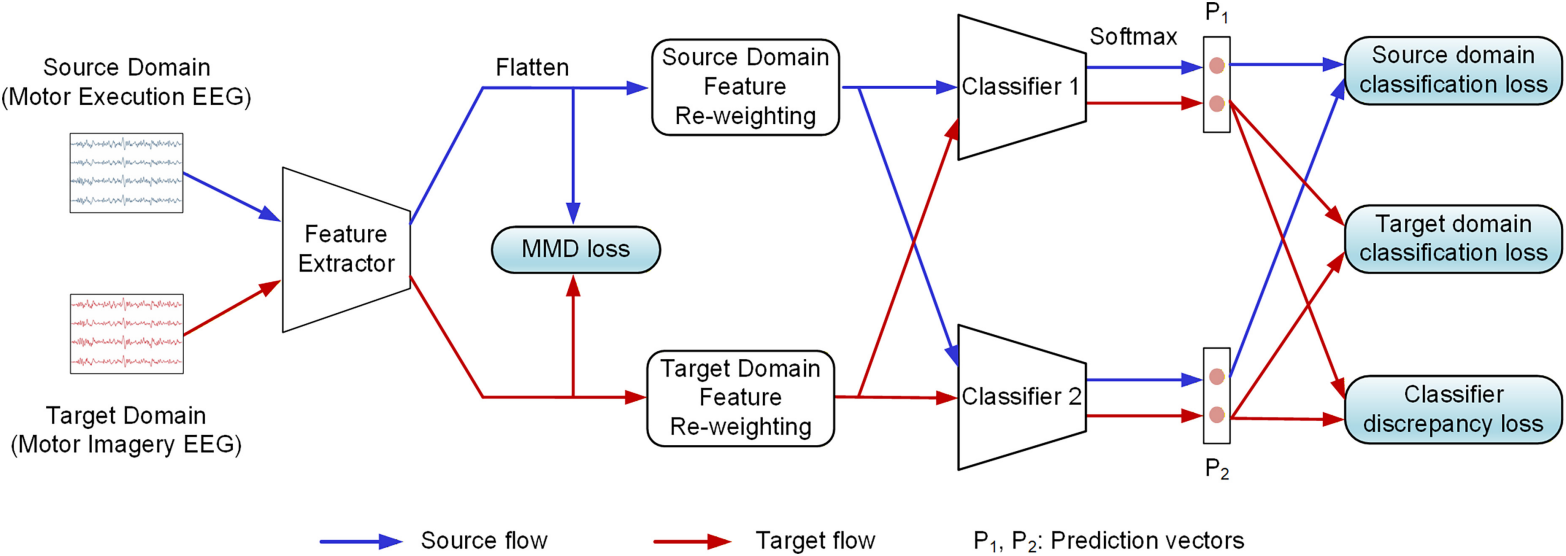
Architecture of the proposed DDCA Net.

Although the similarity between ME and MI motivates the exploration of TL from ME to MI task, differences in their temporal and spatial EEG representations lead to a distribution shift in the feature space. Therefore, DA techniques are required to mitigate the domain shift problem. DA can simultaneously leverage knowledge from the source domain (ME) and the target domain (MI), while aligning their feature representations in a shared space. Accordingly, DA is adopted to address the cross-task TL problem. Specifically, we propose a domain-aware domain–class adaptation network (DDCA Net), which comprises a domain-shared feature extractor, two classifiers, and two domain-specific feature re-weighting (DFR) blocks. As illustrated in Figure 1, domain-level alignment of feature distributions is explicitly achieved by minimizing the maximum mean discrepancy (MMD). Meanwhile, the DFR blocks adaptively emphasize informative features unique to each domain, thereby preserving domain-discriminative characteristics during the alignment process. Furthermore, implicit adversarial alignment is conducted at the class level via bi-classifier learning to encourage consistent decision boundaries between the source and target domains.

The main contributions of this paper are summarized as follows: (1) This study formulates cross-task TL as a domain shift problem and is among the first to address subject-specific TL from ME to MI tasks using a DA paradigm. Accordingly, a novel DDCA Net is proposed to systematically investigate this problem. (2) A hierarchical DA framework is developed, comprising explicit domain-level alignment, implicit class-level alignment, and DFR blocks. These components jointly reduce distribution discrepancies, enforce decision boundary consistency, and preserve domain-specific discriminative knowledge, thereby enabling effective cross-task knowledge transfer. (3) Experimental results demonstrate that DDCA Net maintains strong performance even with limited target-domain data, highlighting its potential to reduce reliance on MI-EEG data in real-world BCI applications.

The remainder of this paper is organized as follows. Section 2 presents the detailed information on the proposed DDCA Net. Section 3 describes the dataset, data preprocessing procedures, experimental settings, and statistical analysis method. The experimental results are reported in section 4, followed by a discussion in section 5. Finally, Section 6 concludes the paper.

## 2 Proposed method

### 2.1 Definitions and notations

We propose the DDCA Net to investigate the feasibility of TL from ME to MI tasks. EEG data collected during the ME task are regarded as the source domain, while EEG data collected during the MI task are regarded as the target domain. Labels from both the source and target domains are utilized during the training process, and the effectiveness of TL is investigated under different proportions of target-domain data used for training. The source domain is denoted as $D_{S}=\left\{X_{S},Y_{S}\right\}=\left\{\left(x_{S}^{1},y_{S}^{1}\right),\ldots ,\left(x_{S}^{n_{S}},y_{S}^{n_{S}}\right)\right\}$. The target domain is denoted as $D_{T}=\left\{X_{T},Y_{T}\right\}=\left\{\left(x_{T}^{1},y_{T}^{1}\right),\ldots ,\left(x_{T}^{n_{T}},y_{T}^{n_{T}}\right)\right\}$. Where $x_{S}^{i}$ and $x_{T}^{i}$ denote the EEG samples in the source and target domains after data augmentation (see section 3.2), respectively, with *E* electrode channels and *T* time points. $y_{S}^{i}$ and $y_{T}^{i}$ represent the corresponding labels of the EEG samples. *n_S_* and *n_T_* denotes the number of samples in the source and target domains.

### 2.2 Network architecture

Figure 1 illustrates the overall architecture of the proposed DDCA Net, which consists of a domain-shared feature extractor (F), two classifiers (C1 and C2), and two DFR blocks, referred to as the source-domain feature re-weighting block R_S_ and the target-domain feature re-weighting block R_T_.

During training, preprocessed EEG samples from both the source and target domains are first fed into F to extract high-dimensional feature representations. These features are then flattened and forwarded to two streams. The first stream performs domain-level alignment by measuring and minimizing the discrepancy between the source- and target-domain feature distributions using the MMD criterion, thereby encouraging the learning of domain-invariant representations.

The second stream is designed to preserve domain-specific characteristics through DFR mechanisms. Specifically, feature representations from the source domain are input to R_S_, whereas those from the target domain are input to R_T_. Each DFR block adaptively emphasizes informative features that are unique to its corresponding domain. The outputs of the R_S_ and R_T_ are then fed into both classifiers, C1 and C2, to produce class predictions for the source and target domains, respectively. By incorporating domain-aware feature refinement, this stream alleviates the negative effects of excessive feature alignment during the adaptation process.

Furthermore, implicit class-level alignment is achieved via a bi-classifier adversarial paradigm, in which the discrepancy between C1 and C2 on target-domain samples is alternately maximized and minimized. This strategy encourages the learning of consistent decision boundaries across domains without requiring explicit class-wise distribution matching.

Finally, the feature extractor F, classifiers C1 and C2, as well as the DFR blocks R_S_ and R_T_, are jointly optimized by minimizing the classification loss on labeled samples from both the source and target domains, enabling the model to learn discriminative and transferable representations across domains.

**FIGURE 2:**
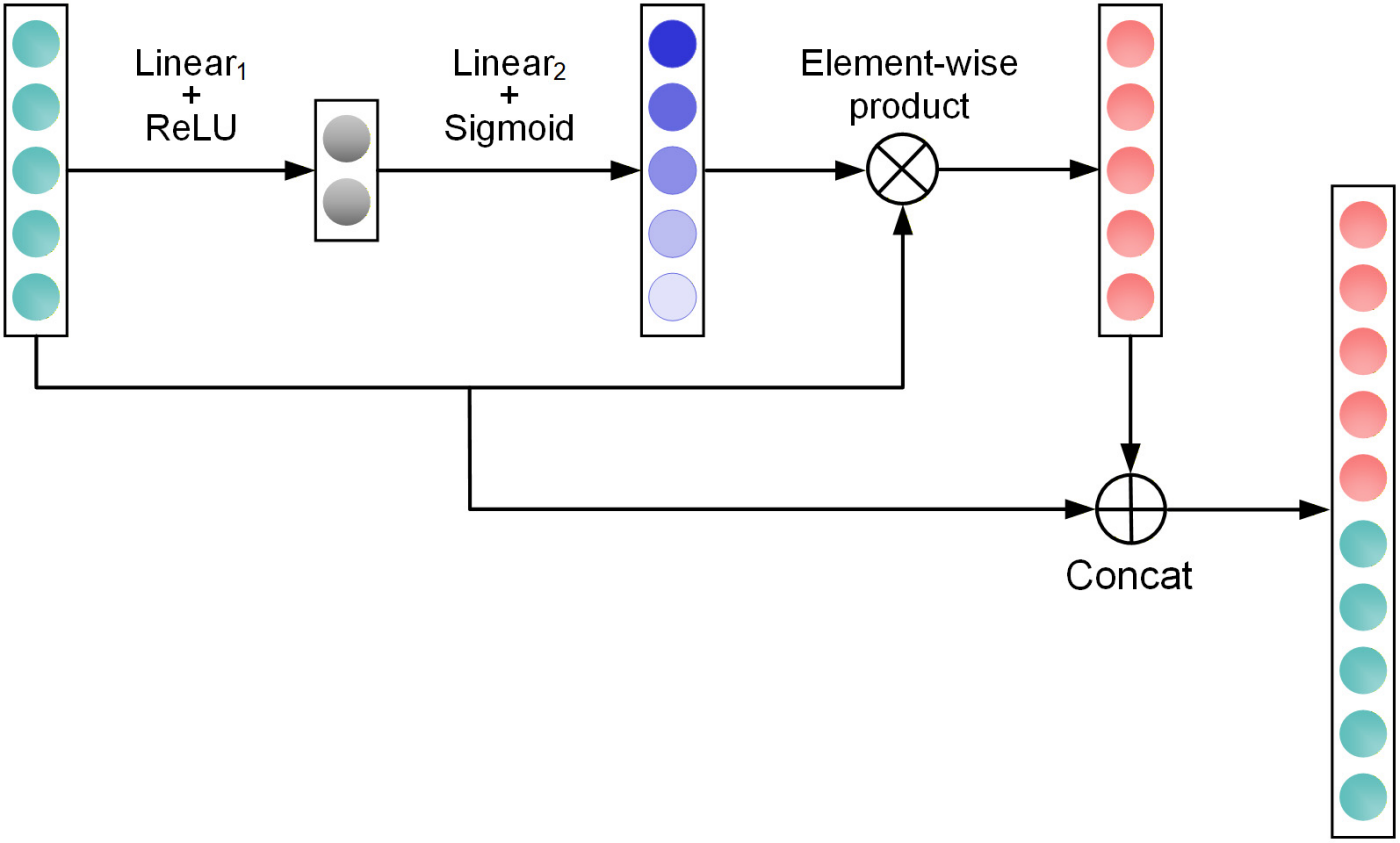
Structure of the DFR block.

Inspired by the SE block, we propose the DFR block to capture temporal–spatial characteristics that are crucial for domain-specific classification. The proposed DFR block operates on flattened temporal–spatial latent features and introduces domain-specific re-weighting, thereby enabling adaptive feature modulation tailored to different domains. The detailed architecture of the DFR block is illustrated in Figure 2. Specifically, the DFR block consists of two fully connected layers. The first layer (Linear1) performs dimensionality reduction using a predefined reduction ratio, which is empirically set to 8 in this study, while the second layer (Linear2) restores the dimensionality of the re-weighting score vector to match the output dimension of the feature extractor. The re-weighting score vector is computed as follows in Equation 1:


$$\begin{array}{c} s=\sigma \,\left({\text{Linear}}_{2}\,\left(\text{ReLU}\,\left({\text{Linear}}_{1}\,\left(f\right)\right)\right)\right) \end{array}$$ (1)

where *f* denotes the output of the feature extractor, σ(⋅) represents the sigmoid activation function, and ReLU(⋅) denotes the rectified linear unit operation. The final re-weighted feature output is obtained according to Equation 2:


$$f^{\prime }=\text{Concat}\,\left(s\,⊗f,f\right)$$ (2)

where *f*’ denotes the re-weighted feature representation produced by the DFR block, ⊗ denotes element-wise product operation, and Concat(⋅) denotes the feature concatenation operation.

To objectively assess the feasibility of cross-task DA, the classification performance of DDCA Net is compared with that of within-task classification (see section 3.3). To focus on the core aspects of the DA method, EEGNet (Lawhern et al., 2018), a widely recognized network architecture, is selected for within-task classification. Accordingly, the convolutional layers of EEGNet are employed as the feature extractor in DDCA Net, while the fully connected layers serve as its classifiers. The detailed structure and parameters of the feature extractor, classifiers, and the DFR blocks are summarized in Table 1.

**TABLE 1:** Model parameters of the feature extractor, classifiers, and the DFR blocks, where *E* is the number of electrode channels and *cls* is the number of classes.

Module	Layers/operations	kernel size	Options
Feature extractor	Conv2D	(1, 32), 8	Mode = same
	BatchNorm		
	DepthwiseConv2D	(*E*, 1), 16	Mode = valid
	BatchNorm		
	ELU		
	AveragePool2D	(1, 4)	
	Dropout		*p* = 0.5
	SeparableConv2D	(1, 16), 16	Mode = same
	BatchNorm		
	ELU		
	AveragePool2D	(1, 8)	
	Dropout		*p* = 0.5
	flatten	160	
DFR block	Linear_1_	20	
	ReLU		
	Linear_2_	160	
	Sigmoid		
	Element-wise product		
	Concat	320	
Classifier	Linear	*cls*	

### 2.3 Optimization objectives

To enable effective cross-task TL from ME to MI, the optimization objectives of the proposed DDCA Net are designed in a hierarchical manner, incorporating both domain-level and class-level alignment. Specifically, the framework is formulated with three complementary objectives: classification learning, domain alignment, and class alignment, which jointly promote comprehensive domain–class adaptation and enhance both the transferability and discriminability of the learned representations.

#### 2.3.1 Classification learning for source and target domains

To establish reliable classification capability, the DDCA Net jointly leverages labeled data from both the source and target domains to learn discriminative representations. For the source domain, labeled EEG data are fed into the feature extractor and classifiers to generate two sets of predictions. The source domain classification loss is computed as the cross-entropy loss between the true labels and the predictions of the two classifiers. By minimizing the source domain classification loss, the model learns transferable knowledge from the source domain. The source domain classification loss can be formulated as follows in Equation 3:


$$L_{S}=\frac{1}{n_{S}}\,\sum_{k=1}^{2}\sum_{i=1}^{n_{S}}L_{c\,e}\,\left(C_{k}\,\left(R_{S}\,\left(F\,\left(x_{S}^{i}\right)\right)\right),y_{S}^{i}\right)$$ (3)

where *L*_*ce*_(⋅) represents the cross-entropy loss, *C*_*k*_(⋅) denotes the label prediction of the *k*-th classifier, *R*_*S*_(⋅) denotes the feature re-weighting operation applied to the source-domain samples, and *F*(⋅) denotes the feature extraction process. Accordingly, the classification loss for the target domain is calculated in Equation 4:


$$L_{T}=\frac{1}{n_{T}}\,\sum_{k=1}^{2}\sum_{i=1}^{n_{T}}L_{c\,e}\,\left(C_{k}\,\left(R_{T}\,\left(F\,\left(x_{T}^{i}\right)\right)\right),y_{T}^{i}\right)$$ (4)

where *R*_*T*_(⋅) denotes the feature re-weighting operation applied to the target-domain samples.

Jointly optimizing classification performance on both domains ensures that the model does not become overly biased toward the source domain, thereby improving generalization to the target domain.

#### 2.3.2 Domain alignment

To achieve global distribution alignment between the two domains, the discrepancy between source- and target-domain feature distributions is minimized using the MMD loss with a Gaussian radial basis function kernel (Hang et al., 2019). Given source-domain features ${\left\{f_{S}^{i}\right\}}_{i=1}^{n_{S}}$ and target-domain features ${\left\{f_{T}^{j}\right\}}_{j=1}^{n_{T}}$, the MMD loss is calculated as shown in Equation 5:


$$\begin{array}{cc} L_{M\,M\,D}=\frac{1}{n_{S}^{2}}\,\sum_{i=1}^{n_{S}}\sum_{j=1}^{n_{S}}k\,\left(f_{S}^{i},f_{S}^{j}\right)+\frac{1}{n_{T}^{2}}\,\sum_{i=1}^{n_{T}}\sum_{j=1}^{n_{T}}k\,\left(f_{T}^{i},f_{T}^{j}\right) & \\ -\frac{2}{n_{S}\,n_{T}}\,\sum_{i=1}^{n_{S}}\sum_{j=1}^{n_{T}}k\,\left(f_{S}^{i},f_{T}^{j}\right) & \end{array}$$ (5)

where *k*(⋅,⋅) denotes the kernel function.

By reducing global domain shift, it facilitates knowledge transfer from ME to MI while serving as a coarse-grained alignment mechanism that provides a foundation for subsequent fine-grained adaptation.

#### 2.3.3 Class alignment

Although the MMD loss explicitly aligns the global feature distributions across domains, it does not account for class-conditional structures. To address this limitation, a bi-classifier adversarial learning strategy is adopted to implicitly align class-level feature distributions by encouraging consistency in decision boundaries across domains. Specifically, two classifiers C1 and C2 are trained to maximize their prediction discrepancy on target-domain samples, and the feature extractor along with two DFR blocks are simultaneously optimized to minimize this discrepancy, thereby pushing target features toward regions where both classifiers agree. To quantify the disagreement, a classifier discrepancy loss is defined on the target domain. The softmax probability for a given sample $x_{T}^{i}$, as predicted by the *k*-th classifier, is calculated in Equation 6:


$$p_{T}^{k}\,\left(x_{T}^{i}\right)=\text{softmax}\,\left(C_{k}\,\left(R_{T}\,\left(F\,\left(x_{T}^{i}\right)\right)\right)\right)$$ (6)

The classifier discrepancy relevance matrix (Kuang et al., 2022) is defined in Equation 7:


$$A\,\left(x_{T}^{i}\right)=p_{T}^{1}\,\left(x_{T}^{i}\right)\cdot {\left(p_{T}^{2}\,\left(x_{T}^{i}\right)\right)}^{\text{T}}$$ (7)

It can be inferred that *A* is a *J* × *J* matrix, where *J* is the number of classification classes. Taken *A*_*mn*_ as the element in the *m*-th row and *n*-th column of *A*, the classifier discrepancy loss is formulated in Equation 8:


$$L_{C\,D}=\frac{1}{n_{T}}\,\sum_{i=1}^{n_{T}}\left(\sum_{m,n=1}^{J}A_{m\,n}-\sum_{m=1}^{J}A_{m\,m}\right)$$ (8)

Through this adversarial process, decision boundary consistency is enforced, which reduces class mismatch and ambiguity across domains and complements the domain alignment to form a unified domain–class adaptation framework.

### 2.4 Training procedure

Following by the training strategy of Maximum Classifier Discrepancy (Saito et al., 2018), a bi-classifier adversarial DA paradigm is adopted in the DDCA Net architecture. Specifically, the extended feature extractor, consisting of the backbone feature extractor and two DFR blocks, is trained together with the two classifiers via a max–min adversarial optimization process. During adversarial TL, the classifier discrepancy loss is alternately maximized and minimized. Both the learning of discriminative representations on the source/target domain and the cross-domain feature alignment are accomplished through a dynamic learning process. The adversarial training procedure is described as follows:

Step 1: Train F, R_S_, R_T_ C1, and C2 by minimizing the classification loss on the source and target domains. The corresponding objective is formulated as follows:


$$\begin{array}{cc} \min_{\theta_{F},\theta_{R_{S}},\theta_{R_{T}},\theta_{C\,1},\theta_{C\,2}}L_{S}\,\left(x_{S}^{i},y_{S}^{i}\right)+L_{T}\,\left(x_{T}^{i},y_{T}^{i}\right)+ & \\ \omega_{M\,M\,D}\,L_{M\,M\,D}\,\left(x_{S}^{i},x_{T}^{i}\right) & \end{array}$$ (9)

Step 2: Optimize C1 and C2 while fixing the parameters of F, R_S_, and R_T_ by maximizing the classifier discrepancy loss. Since F, R_S_, and R_T_ are excluded from the optimization in this step, the classification losses remain unchanged. The objective can be illustrated as follows:


$$\min_{\theta_{C\,1},\theta_{C\,2}}L_{S}\,\left(x_{S}^{i},y_{S}^{i}\right)+L_{T}\,\left(x_{T}^{i},y_{T}^{i}\right)-\omega_{C\,D}\,L_{C\,D}\,\left(x_{T}^{i}\right)$$ (10)

Step 3: Optimize F, R_S_, and R_T_ while fixing the parameters of C1 and C2 by minimizing the classifier discrepancy loss. The corresponding objective is illustrated as follows:


$$\min_{\theta_{F},\theta_{R_{S}},\theta_{R_{T}}}\omega_{C\,D}\,L_{C\,D}\,\left(x_{T}^{i}\right)$$ (11)

In Equations 9–11, ω_*MMD*_ and ω_*CD*_ are trade-off hyperparameters that balance the contributions of domain-level and class-level losses, respectively.

The proposed DDCA Net integrates a domain-aware network architecture with a three-step adversarial training strategy to achieve effective domain–class adaptation. Specifically, the framework consists of a shared feature extractor, DFR blocks, and two parallel classifiers, which collaboratively learn domain-invariant yet class-discriminative representations. The domain-class adaptation is accomplished by repeatedly performing the three-step adversarial training process, in which feature distribution alignment and decision boundary consistency are jointly optimized. For clarity, the overall training and label prediction procedures of the DDCA Net are summarized in Algorithm 1. During the prediction phase, the final predicted label of each sample is determined by combining the outputs of the two classifiers.

Algorithm 1 DDCA Net for ME to MI EEG classification

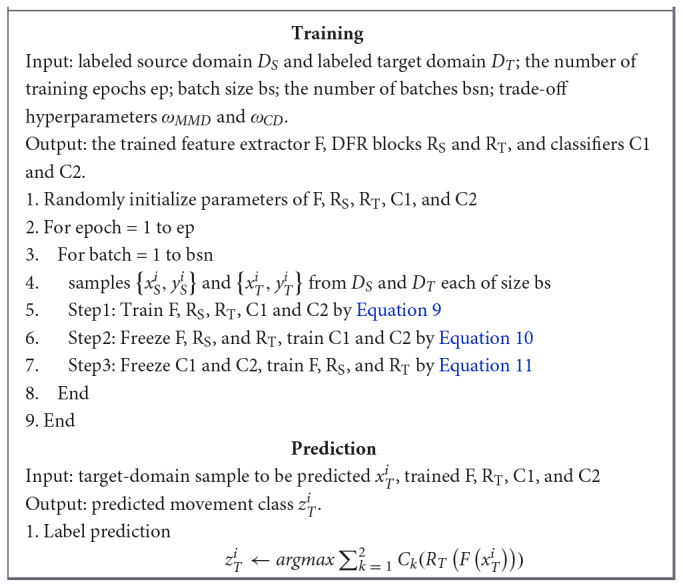

## 3 Experiments

### 3.1 Dataset

In this study, the EEG Motor Movement/Imagery Dataset was employed, which consists of recordings from 109 subjects performing both ME and MI tasks. Each task includes four motor classes: opening and closing of the left fist, right fist, both fists, and both feet. In this work, only the left- and right-fist motor classes from both ME and MI tasks were considered. In addition, two baseline runs were recorded for each subject. EEG signals were acquired using 64 electrodes at a sampling rate of 160 Hz with the BCI2000 instrumentation system (Goldberger et al., 2000; Schalk et al., 2004). The duration of ME/MI task was 4.1s per trial, and approximately 22 trials per class were collected for each subject. Subjects S088, S089, S092, S100, S104 and S106 were discarded from the further analysis due to the nonstandard data structures (Shuqfa et al., 2023), resulting in a final sample of 103 participants.

### 3.2 Data preprocessing

Taking Pz as the reference electrode, the 64-electrode EEG data were re-referenced and band-pass filtered between 8 and 30 Hz. A 4.0-s segment (corresponding to 640 time points) following the instruction onset was extracted as the raw data. To mitigate the issue of limited training data, sliding-window clipping was employed for data augmentation, with a window size of 2.0 s and a step size of 0.25 s. After data augmentation, approximately 200 training samples per class were generated for each subject. Consequently, the dimensions of the augmented data $x_{S}^{i}$ or $x_{T}^{i}$ were 63 × 320 (i.e., *E* electrode channels × *T* time points).

### 3.3 Experiment settings

The DDCA Net model was implemented using PyTorch and trained on an NVIDIA RTX 3090 GPU. The initial learning rate was set to 5 × 10^−4^ and decayed to 5 × 10^−5^ at the 75th epoch, with a total of 150 training epochs. The Adam optimizer was employed with a weight decay of 5 × 10^−4^. The source domain *D*_*S*_ and target domain *D_T_*, as defined in section 2.1, were simultaneously used to train the DDCA Net following Algorithm 1. During training, the batch size for both source- and target-domain samples was set to 24.

To comprehensively validate the feasibility of DA from ME to MI tasks, the performance of DDCA Net was compared with that of within-task classification methods. Specifically, the EEGNet model was trained separately on ME and MI EEG data to perform ME and MI classification, respectively, denoted as ME-EEGNet and MI-EEGNet. Due to the insertion of the DFR module between the feature extractor and the classifiers, the input feature dimension of the classifiers differs from that of EEGNet. Except for this difference, the network architectures and parameter settings of ME-EEGNet and MI-EEGNet follow those listed in Table 1. In this study, MI-EEGNet served as the baseline method, and the classification performance improvement achieved by DDCA Net was systematically investigated.

In the within-task setting, five-fold cross-validation was performed independently for each subject, with 80% of the data used for training and the remaining 20% for testing in each fold. The final within-task performance was obtained by averaging the classification accuracies across the five folds.

For DDCA Net, all labeled samples from the source domain (ME task) were utilized for model training, whereas only a subset of samples from the target domain (MI task) was involved. The proportion of target-domain samples used for training is referred to as the training set ratio (denoted as γ), which was set to 0.5, 0.6, 0.7, and 0.8, respectively. For each subject, the target-domain data were randomly divided into training and testing sets according to the specified γ.

To alleviate the impact of randomness introduced by train–test splitting, each experiment under a given γ was repeated five times, and the average classification accuracy was reported as the final result. Notably, the train–test splitting was conducted prior to sliding-window clipping, ensuring that all testing samples remained completely unseen during model training.

Specifically, the trade-off hyperparameters correspond to domain-level and class-level loss functions, i.e., ω_*MMD*_ and ω_*CD*_, have a significant impact on cross-task TL performance. A grid search is conducted to identify the optimal setting of [ω_*MMD*_, ω_*CD*_] under γ = 0.8. In this process, ω_*MMD*_ is searched from the list [0.001, 0.005, 0.01, 0.05], while ω_*CD*_ is selected from the list [0.005, 0.01, 0.05, 0.1]. The results of the grid search experiment are summarized in Table 2, where it can be observed that the optimal setting of trade-off hyperparameters [ω_*MMD*_, ω_*CD*_] is [0.005, 0.01]. This hyperparameter combination was therefore adopted as the final setting for all subsequent experiments.

**TABLE 2:** Results of the grid search.

**Classification accuracies (%)**		**ω_*MMD*_**			
		0.001	0.005	0.01	0.05
**ω_*CD*_**	0.005	81.42	81.22	81.12	74.86
	0.01	81.32	**81.75**	81.11	73.79
	0.05	81.07	80.79	80.90	73.83
	0.1	81.08	80.88	81.16	74.22

Bold values indicate the optimal hyperparameter combination that achieved the best classification performance.

### 3.4 Statistical analysis

The classification accuracies of DDCA Net under four γ settings and ME-EEGNet are compared with those of the baseline method (MI-EEGNet) to validate the feasibility of cross-task TL. To evaluate the statistical significance of the performance differences, a Friedman test is first conducted to examine overall differences among the six methods (Zhang et al., 2025). Subsequently, *post-hoc* pairwise comparisons between the baseline method and each comparative method are performed using the Wilcoxon signed-rank test with Bonferroni correction. The significance level is set at 0.05.

## 4 Results

### 4.1 Average classification accuracy

The within-task methods and the proposed DDCA Net under four different γ settings were evaluated on each subject in the dataset. The average classification accuracies across 103 subjects for all methods are summarized in Table 3. The Friedman test revealed a statistically significant effect [χ^2^(5) = 211.69, *p* < 0.001], indicating that there were significant differences in performance among the compared methods.

**TABLE 3:** Classification accuracy comparison of intra-task method and the proposed CT-BCADA.

Approch	Method/configuration	Mean (SD)	Median	Range (Max-Min)	*p*
Within-task	MI-EEGNet (Baseline)	74.04 (9.06)	71.97	39.26 (99.02–59.76)	–
	ME-EEGNet	76.17 (9.54)	74.15	39.38 (99.2–59.82)	0.255
DDCA Net	γ= 0.5	73.28 (10.69)	70.34	43.54 (99.11–55.57)	0.377
	γ= 0.6	74.61 (10.44)	72.62	41.95 (99.27–57.31)	1.618
	γ= 0.7	76.52 (10.32)	74.64	41.07 (100.00–58.93)	<0.001
	γ= 0.8	**81.75** (8.92)	79.77	32.68 (99.75–67.07)	<0.001

The classification accuracy represents the average performance across all 103 subjects, expressed in percentage (%). γ denotes the proportion of target-domain samples used for model training. Bold values indicate the highest classification accuracies achieved by the proposed DDCA Net.

The average classification accuracies of MI-EEGNet and ME-EEGNet across 103 subjects were 74.04 and 76.17%, respectively. Although ME-EEGNet achieved a higher accuracy than MI-EEGNet, the improvement was not statistically significant (p = 0.255). For DDCA Net, the average classification accuracy under γ = 0.5 was 73.28%, which was 0.76% lower than that of the baseline. When γ was increased to 0.6, the average accuracy reached 74.61%, representing a marginal improvement of 0.57% over the baseline, but without statistical significance. In contrast, when γ was set to 0.7 and 0.8, the average classification accuracy increased to 76.52 and 81.75%, corresponding to improvements of 2.48 and 7.71% over baseline, respectively. These gains were statistically significant compared with the baseline method (*p* < 0.001).

### 4.2 Subject-level classification accuracy

**FIGURE 3:**
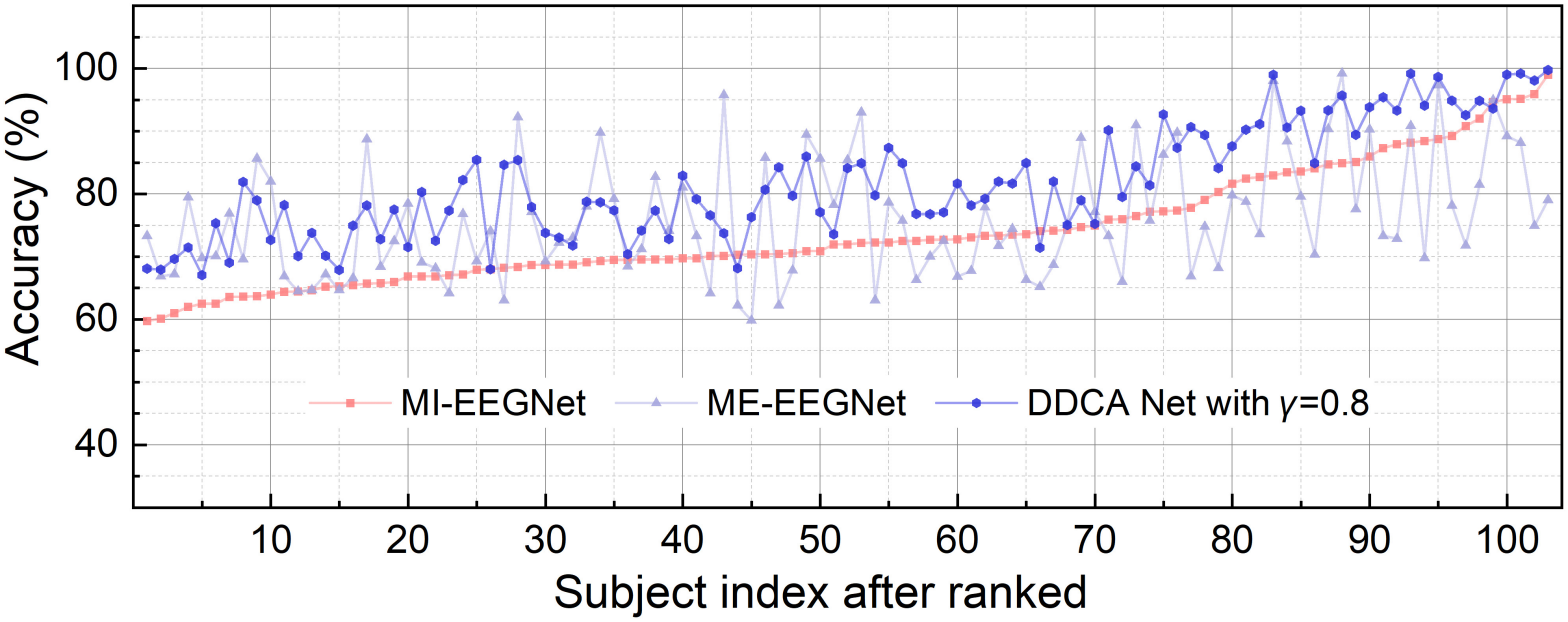
Classification accuracies of different methods for each subject. The subjects are ranked according to their classification accuracies obtained by MI-EEGNet.

Figure 3 illustrates classification accuracies of different methods for each subject. The classification results of MI-EEGNet, ME-EEGNet, and the DDCA Net with γ = 0.8 are presented for comparison. To facilitate a clear comparison of subject-level accuracy, the subjects are ranked according to their classification accuracies obtained by MI-EEGNet. To enhance the clarity of comparison among the three methods, error bars are not displayed in Figure 3. For completeness of statistical information, the corresponding results with error bars (mean ± standard deviation) are provided in Supplementary Figure 1 for reference.

For low-performance subjects (subjects 1–41), whose classification accuracies obtained by MI-EEGNet ranged from 59.76 to 69.74%, 90.24% (37 out of 41) achieved higher classification accuracies with ME-EEGNet than with MI-EEGNet. Within this group, DDCA Net with γ = 0.8 further improved performance, with 65.85% (27 out of 41) of the subjects achieving higher classification accuracies compared with the within-task methods.

For medium-performance subjects (subjects 42–70), where MI-EEGNet accuracies ranged from 70.11 to 74.95%, MI-EEGNet and ME-EEGNet exhibited comparable classification performance. Among these subjects, DDCA Net with γ = 0.8 demonstrated a consistent advantage, with 65.52% (19 out of 29) of the subjects achieving higher classification accuracies than those obtained using the within-task methods.

For high-performance subjects (subjects 71–103), whose MI-EEGNet accuracies ranged from 75.89 to 99.02%, 69.70% (23 out of 33) exhibited lower classification accuracies with ME-EEGNet than with MI-EEGNet, in contrast to the low-performance group. Notably, DDCA Net with γ = 0.8 achieved superior performance for the majority of subjects in this group, with 81.82% (27 out of 33) achieving higher classification accuracies compared with the within-task methods, indicating strong robustness even for subjects with high baseline performance.

**FIGURE 4:**
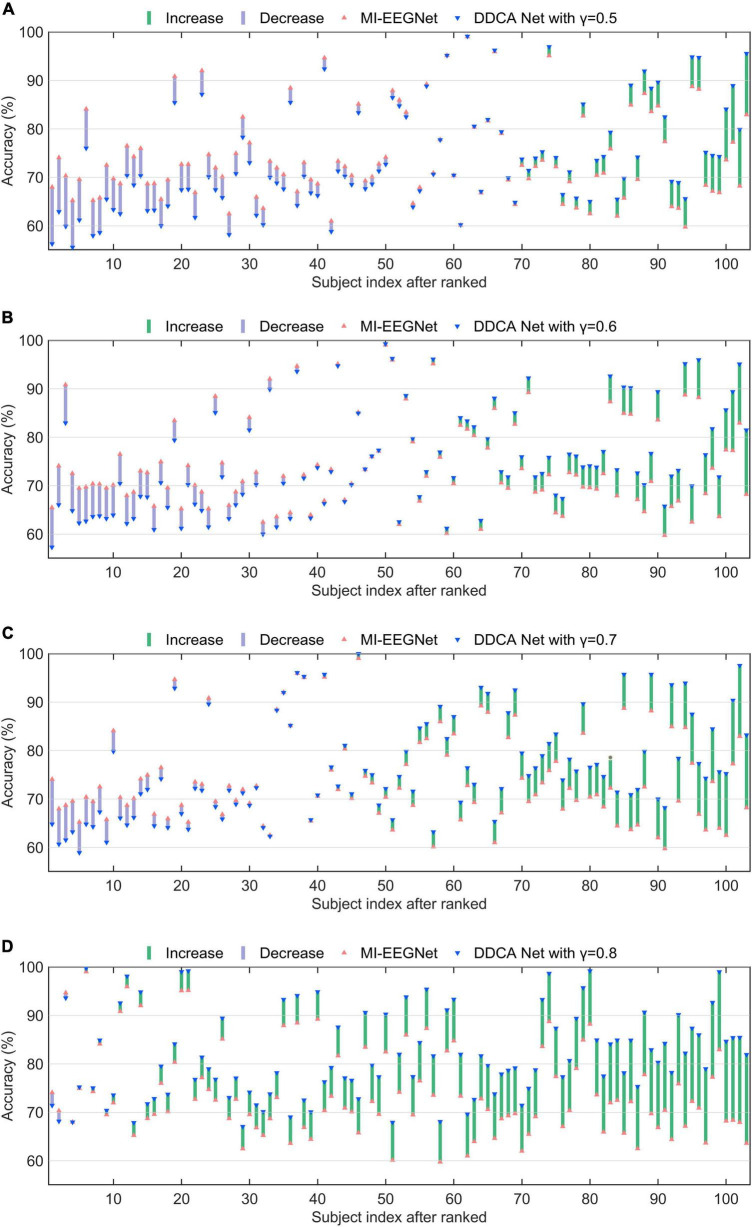
Subject-level comparison of classification performance between the baseline method (MI-EEGNet) and the proposed DDCA Net. Subjects are independently sorted in each subplot according to the performance improvement of the corresponding method, and the x-axis indicates the subject index after ranked. Red triangles denote the classification accuracies of the baseline method, while blue triangles represent the results of DDCA Net with different configurations. Green and light purple bars indicate increases and decreases in accuracy of DDCA Net relative to the baseline, respectively. **(A)** Comparison between MI-EEGNet and the DDCA Net with γ = 0.5. **(B)** Comparison between MI-EEGNet and the DDCA Net with γ = 0.6. **(C)** Comparison between MI-EEGNet and the DDCA Net with γ = 0.7. **(D)** Comparison between MI-EEGNet and the DDCA Net with γ = 0.8.

Figure 4 presents a subject-level comparison of classification performance between the baseline MI-EEGNet and the proposed DDCA Net under different γ values. The proportions of subjects achieving higher classification accuracies with DDCA Net with γ = 0.5, γ = 0.6, γ = 0.7, and γ = 0.8 were 43.69% (45 out of 103), 55.34% (57 out of 103), 66.02% (68 out of 103), and 97.09% (100 out of 103), respectively. When γ = 0.5, fewer than half of the subjects benefited from DDCA Net compared with MI-EEGNet. As γ increased to 0.6 and 0.7, the proportion of subjects showing performance gains exceeded 50%, indicating a progressively improved adaptation effect. Notably, DDCA Net with γ = 0.8 yielded higher classification accuracies for nearly all subjects, consistently improving subject-level performance and exhibiting strong robustness and generalization capability.

In the existing literature, MI-BCI illiteracy is commonly defined by a classification accuracy threshold of 70% (Lee et al., 2019), which corresponds to the low-performance group in this study. As illustrated in Figure 4, the number of MI-BCI illiterate subjects were 41 (baseline), 46 (γ = 0.5), 36 (γ = 0.6), 30 (γ = 0.7), and 8 (γ = 0.8). Remarkably, among the 41 subjects in the low-performance group, 33 achieved classification accuracies exceeding the 70% threshold when using DDCA Net with γ = 0.8, indicating that approximately 80.49% (33 out of 41) of previously BCI–illiterate subjects were converted into literate users.

### 4.3 Visualization analysis of DDCA with varying settings

**FIGURE 5:**
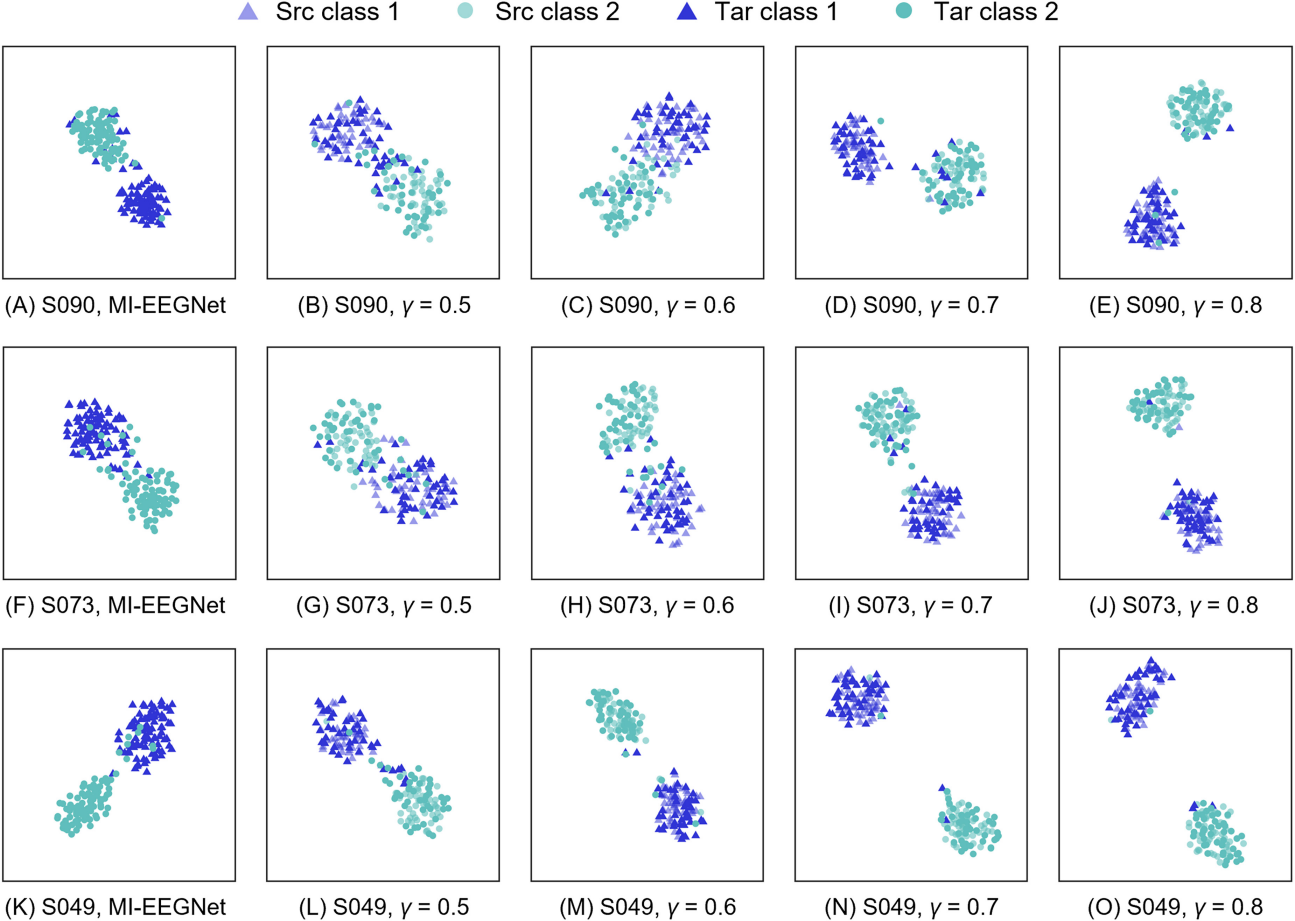
t-SNE visualization of typical subjects, reflecting the impact of γ on classification performance. **(A–E)** Feature distribution for the low-performance subject S090. **(F–J)** Feature distribution for the medium-performance subject S073. **(K–O)** Feature distribution for the high-performance subject S049. Src and Tar denote features from the source and target domains, respectively, while Class 1 and Class 2 correspond to left-hand and right-hand movement classes. “MI-EEGNet” represents the baseline method, while different γ values represent the DDCA method with varying amounts of training MI samples.

To further investigate the domain alignment performance of DDCA Net and demonstrate the effect of γ on classification performance, t-distributed stochastic neighbor embedding (t-SNE) was applied to visualize the high-level representations of different methods for three typical subjects. Subjects S090, S073, and S049 were selected as representatives of the low-, medium-, and high-performance groups, respectively, based on the median or average performance within each group. The t-SNE visualizations shown in Figure 5 revealed that, for all three subjects, the two-dimensional feature space exhibited strong alignment across domains, even under different γ settings. As shown in Figures 5A–E, for S090, when γ was below 0.6, inter-class confusion was observed in both source- and target-domain feature spaces, reducing the discriminability between classes. However, when γ exceeded 0.7 for S090, DDCA Net effectively clustered features within the same class and separated features of different classes, improving classification performance on the target domain. As shown in Figures 5F–O, for S073 and S049, when γ exceeded 0.7, DDCA Net demonstrated superior feature clustering compared to MI-EEGNet. At γ = 0.8, both domains showed exceptional discriminative capacity in the feature space, leading to excellent classification performance on the target domain.

### 4.4 Ablation study

The proposed DDCA Net consists of three modules: domain-level alignment implemented via the MMD criterion, class-level alignment achieved through a bi-classifier adversarial strategy, and a DFR mechanism designed to preserve domain-specific characteristics. To investigate the contribution of each module, an ablation study was conducted under the setting of γ = 0.8.

**FIGURE 6:**
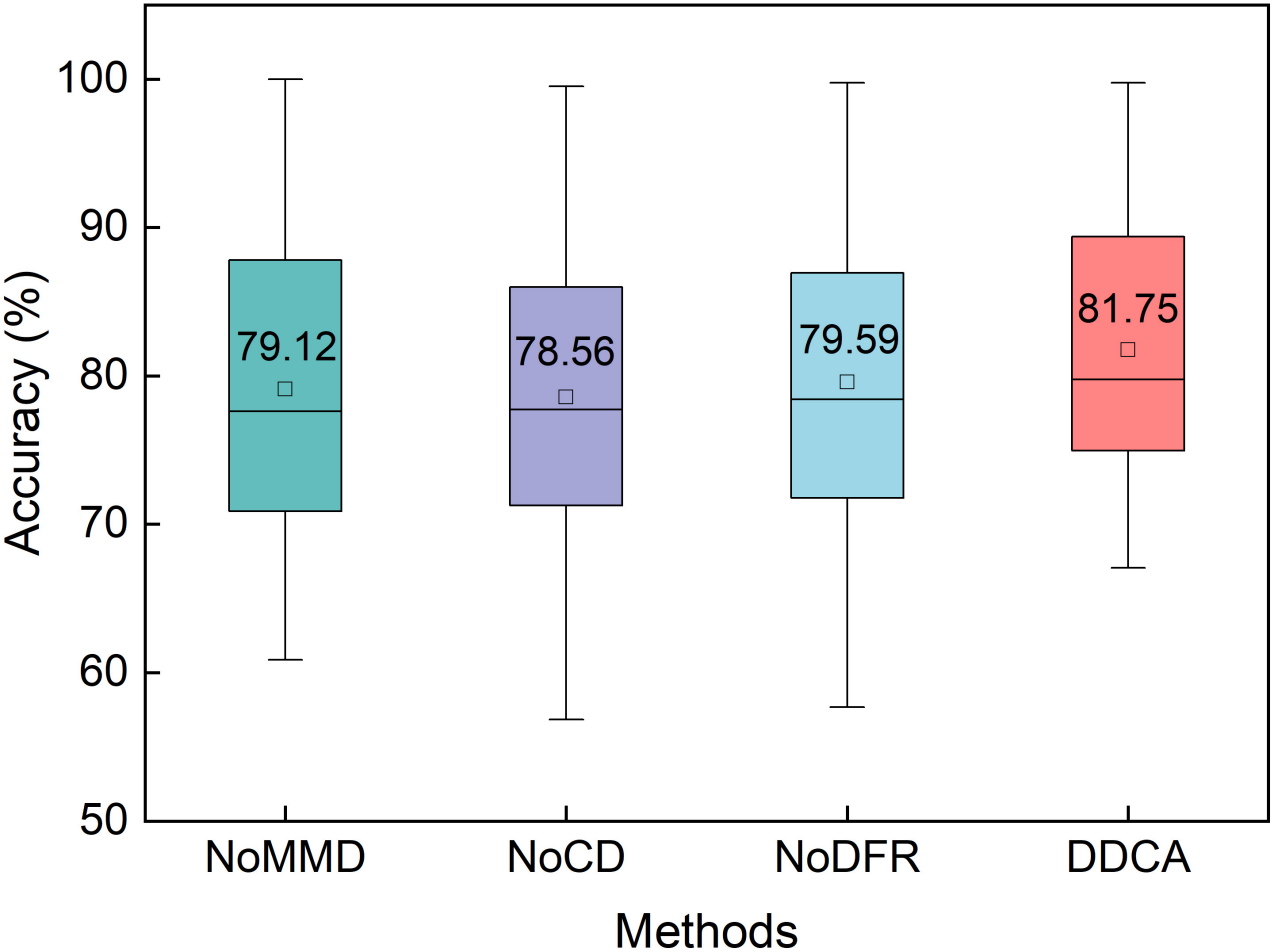
Classification performance of different methods in the ablation study. NoMMD, NoCD, and NoDFR denote variants of DDCA Net without domain-level alignment, class-level alignment, and the DFR mechanism, respectively.

Specifically, three ablated variants were evaluated: DDCA Net without domain-level alignment (NoMMD), without class-level alignment (NoCD), and without the DFR mechanism (NoDFR). Each experiment was repeated five times, and the averaged classification accuracy was reported. The comparative results are illustrated in Figure 6.

As shown in Figure 6, the DDCA Net consistently outperformed all ablated variants, demonstrating the effectiveness of the proposed framework. Among the comparison methods, NoCD achieved the lowest average accuracy, indicating that class-level alignment plays the most critical role in the cross-task DA architecture. Furthermore, compared with the DDCA Net, both NoMMD and NoDFR exhibited performance degradation, suggesting that domain-level alignment and domain-specific characteristic preservation are both beneficial for effective cross-task knowledge transfer in MI-EEG classification.

**FIGURE 7:**
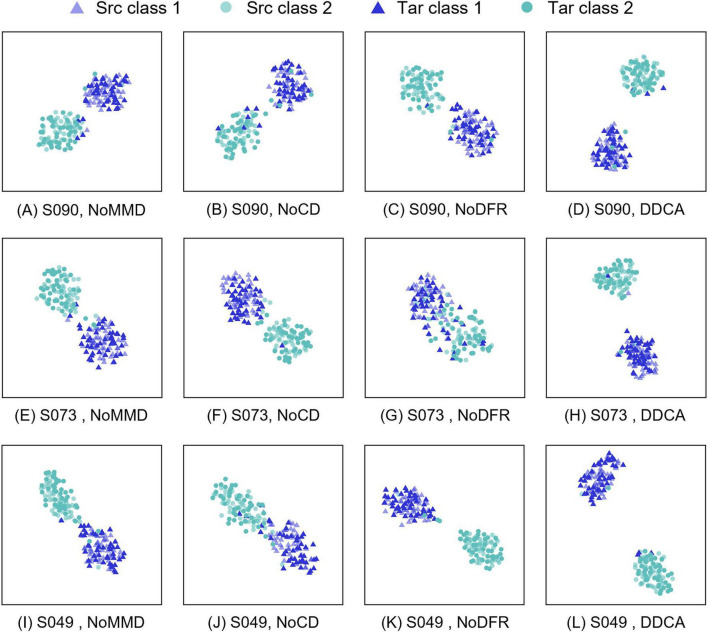
t-SNE visualization of typical subjects for the ablation study, illustrating the contribution of each module in DDCA Net to classification performance. **(A–D)** Feature distribution for the low-performance subject S090. **(E–H)** Feature distribution for the medium-performance subject S073. **(I–L)** Feature distribution for the high-performance subject S049. Src and Tar denote features from the source and target domains, respectively, while Class 1 and Class 2 correspond to left-hand and right-hand movement classes. NoMMD, NoCD, and NoDFR denote variants of DDCA Net without domain-level alignment, class-level alignment, and the DFR mechanism, respectively.

While Figure 6 quantitatively demonstrates the contribution of each module, it does not fully explain the underlying reasons. Therefore, t-SNE visualizations are provided in Figure 7 to offer a more intuitive understanding of how each module influences the feature distribution. Without domain-level alignment (NoMMD), although class-level alignment partially aligned source and target feature distributions, the inter-class separability within each domain was reduced, leading to potential misclassification. Without class-level alignment (NoCD), domain-level alignment alone cannot ensure clear class-wise boundaries, and samples near the decision boundary limited the classification performance. More importantly, without the DFR mechanism, source and target features tended to be overly mixed and the class structure was distorted (e.g., S073 in Figure 7G), indicating that blindly enforcing alignment introduced a trade-off between domains and may cause negative transfer. In contrast, the full model achieved both clear class separation and appropriate alignment while preserving domain-specific structures, demonstrating that DFR mechanism effectively retained domain-specific discriminative information.

## 5 Discussion

In this paper, we explored the feasibility of subject-specific cross-task TL for MI-EEG classification using DA with varying amounts of target-domain training data. The results demonstrated that the proposed DDCA Net outperforms the baseline method, with a 7.71% improvement in classification accuracy and 97.09% of subjects showing enhanced performance when γ was set to 0.8. When γ was reduced to 0.6, the model achieved a modest improvement of 0.57% over the baseline. These findings suggest that DDCA Net effectively facilitates knowledge transfer from the ME task to the MI task by preserving domain-specific characteristics while aligning features at both the domain and class levels.

Figure 3 shows that the proposed DDCA Net exhibits robust and consistently superior performance across subjects with different baseline classification levels. For low-performance subjects, ME-EEGNet generally outperforms MI-EEGNet, indicating that incorporating ME-EEG information is beneficial when the target-domain discriminability is limited. In this scenario, DDCA Net can more effectively leverage discriminative knowledge from the source domain to enhance target-domain classification performance, achieving performance that is comparable to or even higher than that of ME-EEGNet. For medium-performance subjects, DDCA Net demonstrates a complementary effect by adaptively emphasizing informative features from either the source or target domain, thereby compensating for the limitations of within-task methods. As a result, satisfactory classification performance is obtained for the majority of subjects in this group. For high-performance subjects, DDCA Net consistently achieves classification accuracies that exceed those of both MI-EEGNet and ME-EEGNet. This finding suggests that the proposed alignment strategy effectively avoids negative transfer, ensuring that less discriminative feature representations from the source domain do not degrade the target-domain classification performance. Taken together, these results indicate that DDCA Net maintains strong classification performance and robustness across subjects with varying performance levels, highlighting its effectiveness in handling cross-task variability in MI-EEG decoding.

The results presented in Table 3 show that the classification accuracy for the ME task is higher than that for the MI task, which is consistent with previous studies (Tang et al., 2016; Lashgari et al., 2021). However, statistical analysis of the classification performance of ME and MI EEG data using EEGNet revealed no significant difference between the two tasks (number of subjects = 103, *p* = 0.255). A similar phenomenon was also observed in a recent study by Ding et al. (2025). Considering the findings of the present work together with those of Ding et al., the comparable classification performance between ME and MI tasks may be explained as follows: although previous studies have reported greater involvement of the primary motor cortex during ME compared to MI tasks (Miller et al., 2010), DL-based decoders (e.g., EEGNet used in this study) possess a strong ability to automatically learn task-relevant discriminative representations. This capability can narrow the performance gap between ME and MI tasks, thereby resulting in comparable classification performance between the two paradigms.

To further isolate the effect of incorporating source-domain data in the cross-task TL scenario, we conducted an additional cross-domain sample mixing experiment. Specifically, all source-domain data and 80% of the target-domain data were jointly used to train an EEGNet model, while the remaining 20% of the target-domain data were reserved for testing. All other experimental settings followed those described in section 3.3. The results show that the average classification accuracy across all subjects in the sample-mixing setting is 77.16%, which lies between that of MI-EEGNet (74.04%) and DDCA Net with γ = 0.8 (81.75%). This observation indicates that increasing the amount of training data contributes to improve the classification performance; however, the majority of the observed gain can be attributed to the well-designed DA mechanism in DDCA Net, rather than merely the increased data volume. Moreover, the performance improvement achieved by incorporating source-domain data further supports the feasibility and motivation of the cross-task TL problem investigated in this study.

This study investigated the feasibility of cross-task TL using DA and demonstrates the effectiveness of the proposed DDCA Net, thereby filling the gap in this research problem. Compared with the MI task, the ME task offers several advantages. For example, the ME task is less likely to cause fatigue in participants, and the ME task can help participants better perceive muscle movement sensations, thereby enhancing their MI ability. Therefore, integrating ME and MI tasks within a multi-task BCI framework has the potential to reduce the difficulty and fatigue associated with MI training data collection, thereby improving system usability. Notably, DDCA Net with γ = 0.6 achieves a classification performance that is slightly higher than that of MI-EEGNet. Considering that the training set accounts for 80% of the total samples in within-task classification experiments (see section 3.3), it can be inferred that, when adopting the proposed DDCA Net, at least (0.8–0.6)/0.8 × 100% = 25% of MI-EEG trials can be reduced while maintaining comparable performance to the conventional within-task MI-EEG classification approach. These findings demonstrate the practical potential of leveraging DA techniques in cross-task TL scenarios. In particular, they provide empirical support for the development of more efficient and user-friendly BCI systems that integrate ME and MI tasks.

In addition, this study demonstrates that the BCI illiteracy problem can be effectively alleviated through cross-task TL (Figure 4). These results suggest that low-performance subjects can become proficient BCI users when adopting effective decoding strategies, such as cross-task TL, which is consistent with the findings reported in previous studies (Gwon and Ahn, 2024).

While the proposed DDCA Net demonstrated promising results in subject-specific cross-task TL for MI-EEG classification, several limitations exist. One of the practical goals of this study is to reduce the amount of MI-EEG data required for model calibration. However, as shown in Table 3, a relatively high proportion (60–70%) of MI-EEG training data is still required to achieve significant improvements in classification performance. In future work, more sophisticated TL methods should be explored to further reduce the dependence on MI-EEG training data. Compared with existing studies on cross-subject and cross-dataset TL, the present work did not fully exploit the advantages of publicly available datasets or the data-mining capabilities of DL models. Therefore, future research could integrate cross-subject, cross-task, and cross-dataset TL to further improve MI classification performance. In terms of MI paradigms, most existing cross-domain TL studies have focused on the binary classification of left- and right-hand movements (Zhang et al., 2021; Miao et al., 2023; Gwon and Ahn, 2024; Wang et al., 2025) and have primarily examined traditional paradigms involving different limb movements. Few studies have investigated TL classification for fine motor imagery involving different joints of the same limb. However, future BCIs are expected to evolve toward multi-command and intuitive mapping paradigms. Therefore, cross-task TL targeting multiclass and fine motor imagery classification represents a promising direction for future research.

## 6 Conclusion

This study investigated the feasibility of applying DA techniques to TL from ME to MI tasks. The proposed DDCA Net was validated on a public dataset comprising data from more than 100 subjects. The results demonstrated that the proposed DDCA Net achieved 7.71% classification enhancement with 97.09% of subjects exhibiting improved performance compared to the baseline method, confirming the effectiveness of the proposed method. To the best of our knowledge, this work represents one of the first attempts to apply DA techniques to cross-task TL for MI-EEG classification. Building upon this study, future research may explore the applicability of different DA strategies in cross-task, cross-subject, and cross-dataset TL scenarios. Moreover, cross-task TL for MI-EEG classification has the potential to alleviate the difficulty and fatigue experienced by subjects during training protocols, thereby facilitating the practical application of MI-based BCIs.

## Data Availability

Publicly available datasets were analyzed in this study. This data can be found at: https://physionet.org/content/eegmmidb/1.0.0/.
