# Correction: Domain-aware domain–class adaptation network for motor execution to motor imagery EEG classification

**DOI:** 10.3389/fnins.2026.1903945

**Published:** 2026-07-06

**Authors:** Jiahuan Wang, Guanghua Xu, Chenghang Du, Zejin Li, Hui Li, Shengchao Chen, Chengcheng Han, Sicong Zhang

**Affiliations:** 1 School of Mechanical Engineering, Xi'an Jiaotong University, Xi'an, China; 2 State Key Laboratory for Manufacturing Systems Engineering, Xi'an Jiaotong University, Xi'an, China; 3 The First Affiliated Hospital of Xi'an Jiaotong University, Xi'an, China; 4 State Industry-Education Integration Center for Medical Innovations, Xi'an Jiaotong University, Xi'an, China

**Keywords:** brain-computer interface, domain adaptation, electroencephalogram, motor execution, motor imagery, transfer learning

There was a mistake in Table 2 as published. Due to a formatting error, several cells that should have been vertically merged were displayed as separate cells. Specifically, the cell containing “Classification Accuracies (%)” and the cell immediately below it were not merged into a single cell. In addition, the cell containing “ω_CD_” and the three blank cells directly beneath it were not merged into a single cell. The corrected Table 2 appears below.

**Table 2:** Results of the grid search.

Classification accuracies (%)		ω_*****MMD*****_			
		0.001	0.005	0.01	0.05
ω_*CD*_	0.005	81.42	81.22	81.12	74.86
	0.01	81.32	**81.75**	81.11	73.79
	0.05	81.07	80.79	80.90	73.83
	0.1	81.08	80.88	81.16	74.22

Bold values indicate the optimal hyperparameter combination that achieved the best classification performance.

**Equation 10** in **Section 2**, *Section 2.4*, Paragraph 3 was erroneously given as

$\underset{\theta_{C1},\theta_{C2}}{\text{max}}L_{S}\left(x_{S}^{i},y_{S}^{i}\right)+L_{T}\left(x_{T}^{i},y_{T}^{i}\right)-\omega_{CD}L_{CD}\left(x_{T}^{i}\;\right)$.

The correct equation is

$\underset{\theta_{C1},\theta_{C2}}{\text{min}}L_{S}\left(x_{S}^{i},y_{S}^{i}\right)+L_{T}\left(x_{T}^{i},y_{T}^{i}\right)-\omega_{CD}L_{CD}\left(x_{T}^{i}\;\right)$.

**Equation** in **Section 2**, *Section 2.3*, Section 2.3.2, Paragraph 1 was erroneously given as $\{f_{S\;}^{i}i_{=1}^{n_{S}}$. The correct equation is ${\left\{f_{S}^{i}\right\}}_{i=1}^{n_{S}}$.

In the published article, there was an error in the **Section 1**, Paragraph 1, Sentence 3. This sentence previously stated: “Motor imagery (MI) is imaging certain movement of the body part but without any actual motion (Li et al., 2019).” The word “imaging” should be corrected to “imagining”.

A correction has been made to the Section 1, Paragraph 1, Sentence 3:

“Motor imagery (MI) is imagining certain movement of the body part but without any actual motion (Li et al., 2019).”

The original version of this article has been updated.

