# Supplementary material for: Domain-aware domain–class adaptation network for motor execution to motor imagery EEG classification
Source: Front Neurosci. 2026 Jun 1;20:1851006. doi: 10.3389/fnins.2026.1851006 (PMC13265510; doi:10.3389/fnins.2026.1851006)
Supplement: Supplementary file 1 [file Data_Sheet_1.docx]

Supplementary Material

# Supplementary Figures


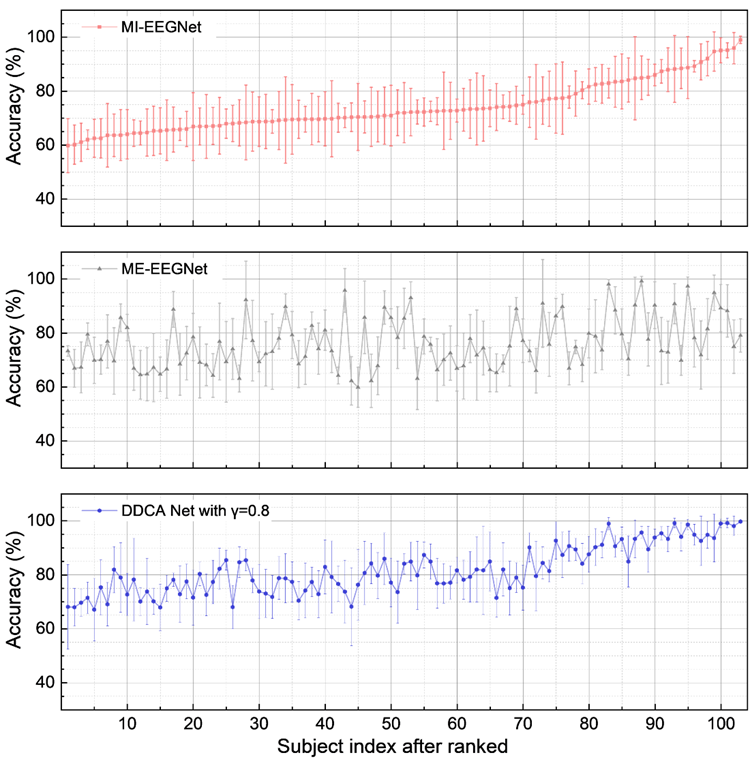


**Supplementary Figure 1.** Classification accuracies of different methods for each subject with error bars (mean ± standard deviation). The subjects are ranked according to their classification accuracies obtained by MI-EEGNet.
